# The Effect of Clavicle Fracture Fixation on Scapular Alignment in Ipsilateral Clavicle and Scapular Fractures

**DOI:** 10.5435/JAAOSGlobal-D-26-00048

**Published:** 2026-07-23

**Authors:** Chittawee Jiamton, Pochong Uthaitas, Wachagorn Kompongpapah, Techit Leelasestaporn, Pariwat Taweekitikul, Thongchai Laohathaimongkol, Theerachai Apivatthakakul

**Affiliations:** From the Department of Orthopaedic Surgery (Jiamton, Uthaitas, Kompongpapah, Leelasestaporn, Taweekitikul, Laohathaimongkol), Queen Savang Vadhana Memorial Hospital, Chonburi, Thailand, and the Department of Orthopedic (Apivatthakakul), Orthopedic Center of Excellence, Bangkok Hospital Chiang Mai, Chiang Mai, Thailand.

## Abstract

**Introduction:**

Ipsilateral clavicle and scapular fractures, often termed “floating shoulder” injuries, represent a severe disruption of the superior shoulder suspensory complex. Although clavicle fixation is standard to restore stability, clinical consensus is lacking regarding its immediate effect on associated scapular displacement. This study aimed to determine whether anatomic fixation of a displaced clavicle immediately improves radiographic alignment of the ipsilateral scapula.

**Methods:**

We conducted a prospective radiographic study of 10 consecutive patients. Intraoperative fluoroscopy (AP and transscapular views) was done immediately before and after definitive clavicular plating. Four alignment parameters were measured: glenopolar angle, medialization, sagittal angulation, and sagittal displacement. Prefixation and postfixation measurements were compared using a paired *t*-test.

**Results:**

No statistically significant differences were found between prefixation and postfixation measurements for any parameter. Mean results (prefixation vs. postfixation) were glenopolar angle (27.24° vs. 27.35°; *P* = 0.954), medialization (28.32 mm vs. 25.71 mm; *P* = 0.153), sagittal angulation (30.06° vs. 30.28°; *P* = 0.948), and sagittal displacement (16.23 mm vs. 14.60 mm; *P* = 0.370).

**Conclusion:**

Surgical fixation of the clavicle alone does not result in notable immediate improvement in radiographic scapular alignment. These findings suggest that scapular malalignment is primarily dictated by the scapular fracture component and associated soft-tissue injury. Surgeons should evaluate the scapular fracture as a distinct entity and not rely on indirect reduction when determining the need for direct scapular stabilization.

The management of the “floating shoulder,” defined as an ipsilateral fracture of the clavicle and scapular neck,^1^ remains debated. This injury represents a double disruption of the superior shoulder suspensory complex (SSSC), which may result in instability and displacement of the glenoid segment.^2^ Although isolated injuries of the SSSC are often treated nonsurgically, combined disruptions have been associated with altered shoulder girdle alignment. Surgical fixation of the clavicle alone has been proposed to restore stability through indirect reduction of the scapular fracture through preserved ligamentous attachments^3^; however, the extent to which this occurs remains unclear. Determining whether clavicle fixation reliably improves scapular alignment is important for guiding surgical decision making and limiting unnecessary surgical exposure.

This study aimed to assess the immediate effect of isolated clavicle fixation on radiographic parameters of scapular alignment in floating shoulder injuries.

## Methods

This study followed a prospective design involving 10 consecutive patients admitted to our trauma center with an acute floating shoulder injury. The diagnosis was confirmed by conventional radiographic and CT evidence of an ipsilateral fracture of the midshaft clavicle and the scapular neck or body. To be included, fractures had to be acute (within 14 days of injury) and demonstrate notable displacement, defined as a clavicle displacement >100% of the shaft width and a scapular neck or body displacement meeting Cole criteria for surgical intervention.^4^ Patients were excluded if they were skeletally immature, had prior shoulder surgery, or had “triple disruptions” involving the acromion or coracoid that would inherently require additional fixation.

### Surgical Intervention

All patients underwent surgical stabilization of the clavicle fracture by a single senior orthopaedic trauma surgeon. The procedure was done under general anesthesia with the patient in the semi–beach-chair position. A standard superior approach for open reduction to the clavicle was used. After exposure of the fracture, anatomic reduction was achieved. Internal fixation was then done using a 3.5 mm precontoured locking compression plate (LCP) and screws.

**Figure 1:**
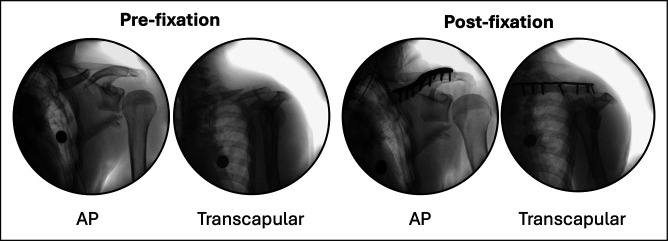
The image demonstrated prefixation and postfixation of scapular in AP and transcapular views (the metal marker was used to calibrate for measurement).

Intraoperative high-resolution fluoroscopy was used to obtain standardized anterior-posterior (AP) scapular (Grashey) and transscapular Y-views. Images were captured at two distinct intervals at the same positioning (Figure 1):

1. Prefixation: After patient positioning but before clavicle reduction.
2. Postfixation: Immediately after definitive clavicle plating.
  After the postfixation assessment, patients who continued to meet surgical criteria underwent subsequent direct scapular stabilization during the same surgical procedure.

### Radiographic Measurements

All radiographic measurements were done digitally using ImageJ (version 1.54) software. To ensure accuracy and minimize bias, two independent investigators (orthopaedic surgeons not involved in the primary procedure) performed the measurements. To calibrate the digital measurements, a metal marker of known diameter was placed within the fluoroscopic field. Interobserver reliability was assessed using intraclass correlation coefficient (ICC).

### Four Primary Parameters Were Measured on the Intraoperative Fluoroscopic Images^5^

**Figure 2:**
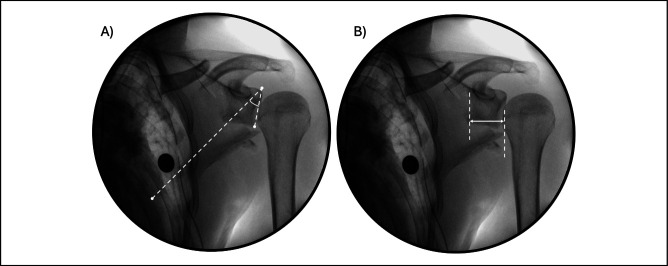
Image showing the parameter in AP view: (**A**) glenopolar angle (GPA) and (**B**) medialization (lateral border offset).

**Figure 3:**
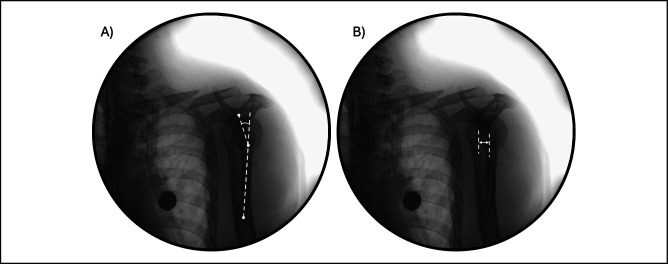
Image showing the parameter in transcapular view: (**A**) sagittal angulation and (**B**) sagittal displacement.

1. Glenopolar angle (GPA): Measured on the AP view as the angle between a line connecting the superior and inferior poles of the glenoid and a line from the superior pole to the scapular apex (Figure 2A).
2. Medialization (lateral border offset): Measured on the AP view as the horizontal distance between parallel vertical lines through the lateral borders of the superior and inferior scapular fragments (Figure 2B).
3. Sagittal angulation: Measured on the transscapular Y-view as the intersection angle of the long axes of the superior and inferior fragments (Figure 3A).
4. Sagittal displacement: Measured on the transscapular Y-view as the distance between the cortical edges of the fragments at the fracture level (Figure 3B).

### Statistical Analysis

Data were analyzed using SPSS version 26. Descriptive statistics (mean and standard deviation) were calculated for all parameters. A paired *t*-test was used to compare prefixation and postfixation measurements, with statistical significance set at *P* < 0.05. To ensure the reliability of the radiographic assessment, interobserver reliability was evaluated using the two-way mixed-effects model for the intraclass correlation coefficient (ICC). After the criteria established by Koo and Li,^6^ ICC values were interpreted as follows: poor (<0.50), moderate (0.50 to 0.75), good (0.75 to 0.90), and excellent (>0.90).

## Results

The study population consisted of 10 consecutive patients. The primary findings of the study are summarized in Table 1, demonstrating the prefixation and postfixation means, standard deviations, and the associated *P*-values.

**Table 1:** Radiographic Parameter of the Scapular Fracture in Prefixation and Postfixation of the Clavicle

Parameter	Prefixation (M ± SD)	Postfixation (M ± SD)	*P*
Glenopolar angle (degree)	27.24 ± 5.42	27.35 ± 3.05	0.954
Medialization (mm)	28.32 ± 11.20	25.71 ± 9.27	0.153
Sagittal angulation (degree)	30.06 ± 9.27	30.28 ± 12.64	0.948
Sagittal displacement (mm)	16.23 ± 9.15	14.60 ± 7.97	0.370

GPA: The mean GPA remained nearly identical, transitioning from 27.24° ± 5.42° prefixation to 27.35° ± 3.05° postfixation (*P* = 0.954). Both values remained below the established physiologic norm of 30° to 45°.

Medialization (lateral border offset): Although this parameter showed the greatest numerical change—decreasing from 28.32 ± 11.20 mm to 25.71 ± 9.27 mm—the reduction of 2.61 mm did not reach statistical significance (*P* = 0.153). Notably, the postfixation mean remained above the 20 mm clinical threshold typically used to indicate direct scapular surgery.

Sagittal angulation: Measurements in the sagittal plane remained stable, with a prefixation mean of 30.06° ± 9.27° and a postfixation mean of 30.28° ± 12.64° (*P* = 0.948).

Sagittal displacement: The mean displacement showed a negligible reduction from 16.23 ± 9.15 mm prefixation to 14.60 ± 7.97 mm postfixation (*P* = 0.370).

The interobserver reliability for all radiographic parameters was assessed to ensure the consistency of the measurements. According to the criteria by Koo and Li,^6^ the overall reliability was found to be moderate to good. The intraclass correlation coefficient (ICC) for the GPA was 0.656 (95% CI, 0.107–0.865), and for medialization, it was 0.734 (95% CI, −0.181 to 0.922). For parameters measured on the transscapular Y-view, the ICC for sagittal angulation was 0.758 (95% CI, 0.285 to 0.910), and for sagittal displacement, it was 0.667 (95% CI, −0.157 to 0.890). These values confirm that the intraoperative fluoroscopic measurements provided a reliable basis for the study's conclusions.

## Discussion

The management of floating shoulder injuries has evolved from nonsurgical treatment to surgical stabilization strategies^3,7-9^ Isolated clavicle fixation has been advocated as a less invasive alternative to dual fixation,^10,11^ based on the concept that restoring the superior strut of the superior shoulder suspensory complex (SSSC) allows indirect reduction of the scapula. This study challenges this assumption.

Our findings demonstrate that clavicle fixation alone does not result in a statistically significant immediate improvement in any of the four evaluated radiographic parameters of scapular alignment, including the glenopolar angle (GPA), medialization, sagittal angulation, and sagittal displacement. These results contrast with previous reports^3^ suggesting improved scapular alignment after clavicle fixation and indicate that the mechanical coupling between the clavicle and the displaced scapular segment may be limited.

Scapular displacement in double disruptions of the SSSC is influenced by gravitational forces and muscular vectors acting on the coracoid-based distal fragment. Restoration of clavicular length alone is insufficient to counteract these forces, particularly when ligamentous structures are compromised or fracture comminution permits continued displacement. Accordingly, indirect reduction through clavicle fixation may not reliably occur.

Although previous clinical series have reported satisfactory functional outcomes after isolated clavicle fixation,^10-12^ many lack radiographic validation of scapular alignment. This study provides the missing intraoperative link, demonstrating that scapular malalignment persists after clavicle plating, suggesting that favorable outcomes may reflect patient compensation rather than anatomic restoration. However, such compensation rather than anatomic restoration has clinical limits. Persistent medialization (>20 mm) and a low GPA (<20°) are strongly associated with long-term abductor weakness, fatigue, and rotator cuff dysfunction.

Because clavicle fixation alone does not correct these deformities, the scapular fracture must be considered a distinct pathologic entity. Our findings indicate that scapular malalignment is driven primarily by the scapular fracture and associated soft-tissue injury rather than by a loss of clavicular support alone. This aligns with more recent comparative studies, such as those by Lin et al,^7^ Egol et al,^13^ and Shao et al,^9^ which suggest that dual fixation leads to superior anatomic and functional outcomes compared with isolated fixation or conservative management. Although Herscovici^14^ and others^11,12^ reported success with clavicle-only fixation, our data provide a biomechanical explanation for why that success may be inconsistent or dependent on initial low-energy fracture patterns. In markedly displaced injury, the internal forces of the shoulder girdle are simply too great for indirect reduction to be effective.

The failure of indirect reduction underscores the importance of the soft-tissue envelope in floating shoulder injuries. As described by Goss, the SSSC functions as a bone-ligament ring, and disruption of its ligamentous components renders the system unstable. Accordingly, management strategies should be reconsidered. Rather than assuming secondary scapular reduction after clavicle fixation, surgeons should independently assess scapular stability and alignment. If the glenopolar angle remains below 20° to 30° after clavicle fixation, direct scapular stabilization should be considered.

Our study used intraoperative fluoroscopy, which is a standard tool for surgical decision making. Although CT is superior for precise preoperative measurement,^5^ fluoroscopy provides the necessary real-time feedback during the procedure. The extreme lack of change between prefixation and postfixation time points (*P*-values ranging from 0.153 to 0.954) provide strong evidence for our conclusions.

The GPA, in particular, remained almost identical (27.24° vs 27.35°). This is critical because the GPA is the most reliable predictor of functional outcomes in scapular neck fractures.^7^ If a surgeon's goal is to restore the normal spatial relationship of the glenoid to the scapular body, clavicle fixation alone is clearly an insufficient tool. Our findings suggest that the reliance on “indirect reduction” through clavicle fixation is often insufficient to restore scapular anatomy. Therefore, we propose a modification to the standard surgical algorithm for floating shoulder injuries. Surgeons should prioritize an independent intraoperative assessment of the scapula immediately after definitive clavicle plating. If the GPA remains below the physiologic norm of 20° to 30°, or if medialization exceeds 20 mm, clinicians should not expect spontaneous improvement. In such cases, direct scapular stabilization should be done during the same anesthetic episode to prevent long-term abductor fatigue and rotator cuff dysfunction.

Based on the findings of this study, a modification of the surgical algorithm for floating shoulder injuries is proposed:

1. Independent assessment: Scapular fracture displacement must be evaluated independently of the clavicle. If the scapula meets established surgical criteria, direct fixation should be planned as the primary goal.
2. Avoid reliance on indirect reduction: Surgeons should not expect clavicle fixation alone to improve scapular alignment because intraoperative “secondary reduction” is unlikely.
3. Stepwise surgical strategy: Although clavicle fixation may be done first to restore the superior strut, immediate intraoperative reassessment of scapular alignment is essential. If malalignment persists, direct scapular fixation should be done during the same anesthetic episode when clinically appropriate.
4. Patient counseling: Patients should be counseled that clavicle fixation alone may not correct scapular displacement and that dual fixation may be required to optimize functional outcomes.

This study has several limitations. First, the sample size of 10 patients is relatively small, which may limit the generalizability of the findings to all fracture patterns. However, floating shoulder injuries are rare, and a cohort of 10 consecutive, prospectively analyzed patients provides notable insight into immediate radiographic changes. Second, the study focused only on immediate radiographic alignment and did not include long-term functional follow-up or a comparison group for dual fixation. Nevertheless, our objective was specifically to test the immediate “indirect reduction” theory, which our data successfully addressed. Although previous literature suggests that patients may achieve satisfactory outcomes through muscular compensation, our data clarify that such outcomes are likely achieved despite persistent scapular malalignment, rather than because of its correction. Finally, intraoperative fluoroscopy can be limited by patient positioning; however, we used standardized AP and transscapular Y-views to minimize error, and the consistency of the findings across multiple parameters suggest the results are robust.

## Conclusion

Surgical reduction and fixation of the clavicle fracture alone does not lead to a statistically significant immediate improvement in radiographic scapular alignment in patients with ipsilateral clavicle and scapular fractures. These findings suggest that the scapular fracture component and associated soft-tissue injury are the primary factors dictating scapular malalignment, and that the routine management of these combined injuries may warrant re-evaluation regarding the need for direct scapular stabilization.

## References

[1] Ganz R Noesberger B: Treatment of scapular fractures. Hefte Unfallheilkd 1975:59-62. 1234274

[2] Goss TP: Double disruptions of the superior shoulder suspensory complex. J Orthop Trauma 1993;7:99-106. 8459301 10.1097/00005131-199304000-00001

[3] Yadav V Khare GN Singh S : A prospective study comparing conservative with operative treatment in patients with a “floating shoulder” including assessment of the prognostic value of the glenopolar angle. Bone Joint J 2013;95-B:815-819. 23723278 10.1302/0301-620X.95B6.31060

[4] Cole PA Gauger EM Schroder LK: Management of scapular fractures. J Am Acad Orthop Surg 2012;20:130-141. 22382285 10.5435/JAAOS-20-03-130

[5] Anavian J Conflitti JM Khanna G Guthrie ST Cole PA: A reliable radiographic measurement technique for extra-articular scapular fractures. Clin Orthop Relat Res 2011;469:3371-3378. 21360211 10.1007/s11999-011-1820-3 PMC3210266

[6] Koo TK Li MY: A guideline of selecting and reporting intraclass correlation coefficients for reliability research. J Chiropr Med 2016;15:155-163. 27330520 10.1016/j.jcm.2016.02.012 PMC4913118

[7] Lin TL Li YF Hsu CJ : Clinical outcome and radiographic change of ipsilateral scapular neck and clavicular shaft fracture: Comparison of operation and conservative treatment. J Orthop Surg Res 2015;10:9. 25626962 10.1186/s13018-014-0141-0 PMC4314750

[8] Edwards SG Wood GW 3rd Whittle AP: Factors associated with short Form-36 outcomes in nonoperative treatment for ipsilateral fractures of the clavicle and scapula. Orthopedics 2002;25:733-738. 12138959 10.3928/0147-7447-20020701-13

[9] Shao Y Zhu X Liu B Ji C Sun J Chen G: Is fixation of both clavicle and scapula better than clavicle alone in surgical treatment of floating shoulder injury? A retrospective study. BMC Musculoskelet Disord 2023;24:605. 37491231 10.1186/s12891-023-06583-8 PMC10367396

[10] Hashiguchi H Ito H: Clinical outcome of the treatment of floating shoulder by osteosynthesis for clavicular fracture alone. J Shoulder Elbow Surg 2003;12:589-591. 14671523 10.1016/s1058-2746(03)00179-4

[11] Samy MA Darwish AE: Fixation of clavicle alone in floating shoulder injury: Functional and radiological outcome. Acta Orthop Belg 2017;83:292-296. 30399993

[12] Gilde AK Hoffmann MF Sietsema DL Jones CB: Functional outcomes of operative fixation of clavicle fractures in patients with floating shoulder girdle injuries. J Orthop Traumatol 2015;16:221-227. 25940307 10.1007/s10195-015-0349-8 PMC4559540

[13] Egol KA Connor PM Karunakar MA Sims SH Bosse MJ Kellam JF: The floating shoulder: Clinical and functional results. J Bone Joint Surg Am 2001;83:1188-1194. 11507127 10.2106/00004623-200108000-00008

[14] Herscovici D Jr Fiennes AG Allgower M Ruedi TP: The floating shoulder: Ipsilateral clavicle and scapular neck fractures. J Bone Joint Surg Br 1992;74:362-364. 1587877 10.1302/0301-620X.74B3.1587877

